# Blockade of fast A-type and TEA-sensitive potassium channels provide an antiparkinsonian effect in a 6-OHDA animal model

**DOI:** 10.17712/nsj.2017.1.20160266

**Published:** 2017-01

**Authors:** Hashem Haghdoost-Yazdi, Hossein Piri, Reza Najafipour, Ayda Faraji, Negin Fraidouni, Tahereh Dargahi, Mahmud Alipour Heidari

**Affiliations:** *From the Student Research Committee (Faraji, Fraidouni, Dargahi), School of Medicine, and from the Cellular and Molecular Research Center (Haghdoost-Yazdi, Piri, Najafipour, Alipour Heidari), Qazvin University of Medical Sciences, Qazvin, Iran*

## Abstract

**Objective:**

To evaluate the effect of K^+^ channels inhibitors in treatment of parkinson’s disease (PD).

**Methods:**

This prospective comparative study was conducted in the Qazvin University of Medical Sciences, Iran, from April 2015 to January 2016. Male rats (n=37) received intraperitoneal doses of TEA (2 and 5 mg/kg) or 4-AP (0.5 and 1 mg/kg) twice-daily, before a stereotactic injection of 6-hydroxydopamine (6-OHDA) for the following 7 days. The 6-OHDA was injected into right medial forebrain bundle (MFB) of the rat brains. Development and severity of PD were assessed using the apomorphine-induced rotational test, the elevated body swing test and rotarod tests. Concentration of malondialdehyde (MDA), a marker of oxidative stress, was measured in rat sera.

**Results:**

Tetraethylammonium and 4-AP significantly reduced the number of apomorphine-induced rotations and improved motor learning in the rotarod test at both doses. Administration of 4-AP and TEA together was more effective than single administration of either agent. Malondialdehyde measurement showed that pretreatment with TEA could not prevent 6-OHDA-induced oxidative stress.

**Conclusion:**

Our results showed that pretreatment with TEA and 4-AP has a neuroprotective effect against 6-OHDA in dopaminergic neurons in the substantia nigra.

Parkinson’s disease (PD) is a prevalent disorder of the nervous system. The main pathophysiologic cause of this disease is a decrease in activity, or death, of dopaminergic neurons of the substantia nigra (SN) pars compacta. There is currently no cure for PD, but the optimal available treatment is L-dihydroxyphenylalanine (L-DOPA). Although the discovery of L-DOPA revolutionized the treatment of the disease, and ameliorates patients’ motor impairments, its effect decreases after 4 or 5 years, and patients suffer from dyskinesia, which diminishes their quality of life. However, recent studies have focused on the discovery of new methods to prevent both the death of dopaminergic neurons and progression of PD.^1^ Potassium (K^+^) channels are the most diverse type of ion channel in all living cells, and play a major role in controlling the electrical activities of both neurons and signaling pathways, which regulate neuronal life and death. It has also been shown that K^+^ channels play a pivotal role in regulating the activity of enzymes and caspases that lead to neuronal apoptosis^2^,^3^ and that amplification of extracellular K^+^ currents and reduction of intracellular K^+^ concentrations mediated by over activation of voltage-gated K^+^ channels are important steps in apoptosis.^2^-^4^ In apoptotic immune and nervous cells, the concentration of intracellular K^+^ ions decreases noticeably, leading to activation of caspase 3 and apoptosis.^4^

Delayed rectifier K^+^ channels are over expressed during some particular apoptotic levels of many apoptotic factors in cholinergic septal cells and cortical section neurons.^5^ Tetraethylammonium (TEA) and 4-aminopyridine (4-AP) are potent inhibitors of K^+^ channels. Tetraethylammonium is an organic compound that blocks delayed rectifier and large conductance Ca^2+^-dependent K^+^ channels and in this manner inhibits apoptotic cell death and also increases neuron excitability of neurons, resulting in the firing of action potentials.^3^,^4^ Recent studies have shown that TEA and its analogues reduced all apoptotic features in thymocyte cells in micromolar concentrations.^6^ With regard to the effect of TEA on the cytoplasmic surface of voltage-dependent channels, the inductive effect of staurosporine (which activates caspase-3), led to reduction of neuronal apoptosis. The 4-AP is a powerful blocker that inhibits an extensive range of K^+^ channels, particularly fast-inactivating K^+^ channels that mediate A-type current.^3^,^4^ By inhibiting these channels, 4-AP abrades deactive neurons, converting the firing pattern of the action potential from the tonic state to the detonation state. For example, in the Purkinje cells of the cerebellum, the usage of 4-AP allows quiescent neurons to become active and amplify the activities of other neurons.^7^,^8^ The 4-AP improves neurologic disorders that are the result of abnormal activity of Purkinje cells.^9^,^10^ Previously, we assessed the effect of 4-AP and TEA in the treatment of 6-hydroxydopamine (6-OHDA)-induced parkinsonism in rats. We hypothesize that these types of K^+^ channel-blockers can reduce the symptoms of this parkinsonism by an increase in the electrical activity of dopaminergic neurons in the SN.^11^

Here, in this study, we hypothesized that 4-AP and TEA have neuroprotective effect through decrease in K^+^ currents and progression of apoptosis and inhibition of many of the enzymes that promote cell death signaling. To test this hypothesis, we evaluated the effect of pretreatment with these agents on the severity of behavioral symptoms of 6-OHDA-induced parkinsonism. In order to do this, 4-AP and TEA were administered twice daily before stereotactic injection of 6-OHDA in the following 7 days.

## Methods

**Figure 1:**
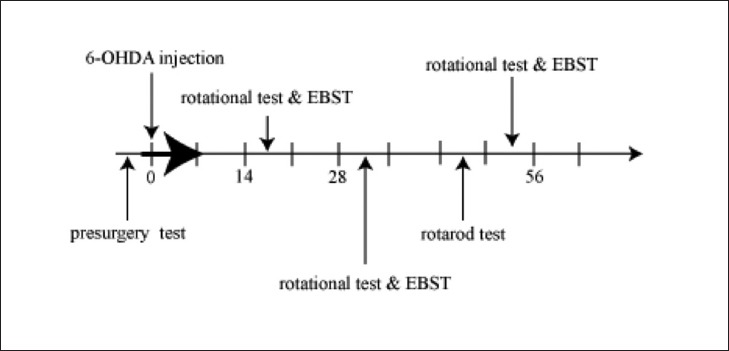
- Time schedule used for our experiments: Animals were tested by apomorphine-induced rotational test and elevated body swing test (EBST) at fourth times: before stereotaxic surgery and 6-OHDA injection and in the third, fifth and eighth weeks after that. Rotational tests were performed at least 1 hour after termination of the EBST. Rotarod test, were performed in the seventh week after 6-OHDA injection. Blood sampling and measurement of its MDA concentration were performed before the surgery and in the fourth and ninth weeks after that. Pretreatments with potassium channel blockers of 4-AP and TEA and also saline was begun just before 6-OHDA injection and continued to seven days after that (black arrow). Numbers show the days after 6-OHDA injection. EBST - elevated body swing test, MDA - Malondialdehyde, 4-AP - 4-aminopyridine, TEA - Tetraethylammonium, 6-OHDA - 6-hydroxydopamine

This prospective, comparative study was conducted in the Cellular and Molecular Research Center, Qazvin University of Medical Sciences, Qazvin, Iran, from April 2015 to January 2016. The 4-AP, TEA, 6-OHDA, and apomorphine were bought from Sigma-Aldrich, and 6-OHDA and apomorphine were prepared on a daily basis. The 4-AP and TEA were dissolved in normal saline. Adult male Wistar rats (n=45) were divided into 6 experimental groups as follows: veh (n=8), which received 0.1 ml saline as a solvent of TEA and 4-AP; low 4-AP received 4-AP at a dose of 0.5 mg/kg (n=8) and high 4-AP received 4-AP at a dose of 1 mg/kg (n=7), low TEA (n=8) received TEA at a dose of 2 mg/kg and high TEA (n=7) received TEA at a dose of 5 mg/kg, and 4-AP+TEA (n=7) received a combination of 4-AP and TEA at doses of 1 and 5 mg/kg, respectively. All pretreatments were intraperitoneally injected 30 minutes before 6-OHDA injection, and then every 12 hours for the following 7 days (**Figure 1**). Data from another group of rats (n=8), marked as healthy rats, which did not undergo surgery and did not receive 6-OHDA, were used to analyze the rotarod test.

### Surgical procedures

First, all rats (with the exception of the healthy group) were anesthetized using an intraperitoneal injection of a solution containing ketamine (100 mg/kg) and xylazine (5 mg/kg). The 6-OHDA was then injected into the right medial forebrain bundle (MFB) near to the SN, using stereotactic apparatus (Stoelting, Wood Dale, IL 60191, USA). The coordinates for the injections were AP: -4, L: -0.8, DV:-8 and AP: -4.4, L: -1.2, DV: -7.8. AP and L were measured from the bregma and DV was measured from the surface of skull, according to the atlas of Paxinos and Watson,^12^ and through a 10-µl Hamilton syringe. The 6-OHDA was dissolved in normal saline containing 0.2 mg/ml of ascorbic acid, and was injected over a 5-minute period. Following injection, the needle was left in place for an additional 5 minutes and then withdrawn.

### Behavioral testing

The animals were tested four times using the apomorphine-induced rotational test and elevated body swing test (EBST): before stereotactic surgery and 6-OHDA injection, and then in Weeks 3, 5, and 8 following injection. Rotational tests were performed at least 1 hour after termination of the EBST. The rotarod test was performed at Week 7 after 6-OHDA injection. Pretreatments with 4-AP and TEA, as well as saline, were begun immediately prior to the 6-OHDA injection and continued for additional 7 days (**Figure 1**).

### Apomorphine-induced rotational test

The apomorphine-induced rotational test was carried out according to the procedure described by Fujita et al.^13^ The animals received intraperitoneally injected apomorphine hydrochloride (0.5 mg/kg, dissolved in saline), after which we counted the number of full rotations in a cylindrical glassy container at 10-minute intervals for 1 hour. Contralateral and ipsilateral rotations (far away in the contralateral and toward the lesioned side in the ipsilateral rotation) were counted as positive and negative scores, and the net number of rotations was defined as the positive minus the negative scores.

### Elevated body swing test

The EBST was conducted prior to the apomorphine-induced rotational test. In brief, each animal was held in a vertical position, 2 cm away from the base of its tail. It was then elevated to 2 cm above the surface on which it was resting. Once the animal was held in the vertical axis, we counted the number of swings to the right or left for 1 minute. One observer held the rat, while another was responsible for recording the direction and frequency of swings. One biased swinging behavior was calculated as follows: L/(L+R) (%) for left-biased swings and R/(R+L) (%) for right-biased swings (L=amount of left-biased swings, R= amount of right-biased swings).

### Rotarod test

The rotarod test evaluated movement coordination, learning ability, and maintenance of the balance of the rats. This test was performed on 3 consecutive days and in 2 sessions per day. Each session lasted 120 seconds, during which the rotating rod underwent a linear acceleration from 5 to 40 rpm. The amount of time that the animals were capable of stepping on the rotating rod was a criterion of their performance ability. The rotarod data were expressed as the area under the curve (AUC), which was computed according to the following formula: AUC= time on the rod (s) × [time on the rod(s) × 0.44/2] where 0.44 is the acceleration speed per second.

### Blood sampling and MDA measurement

MDA concentration measurement as an oxidative stress marker was taken at three steps: before 6-OHDA injection, and then 21 and 60 days after injection. Blood samples were taken from the animals’ tails. This test was performed using the colorimetric method described by Albro et al.^14^ Sera were separated from the clotted blood by centrifugation. A thiobarbituric acid (TBA) and MDA standard curve was used for measurement of MDA concentrations, since MDA and TBA react together to produce a pink-colored solution with 532 nm maximum light absorbance.

### Statistical analysis

Data were expressed as the mean±SD, despite the probable non-normality of the distribution of scores. The behavioral tests data and the MDA assay results were initially analyzed using the Kolmogorov-Smirnov test to find the normality of the data. Since these data lacked normal distribution, they were subsequently analyzed using Kruskal-Wallis nonparametric analysis of variance followed by a two-tailed Mann–Whitney U test. A *p* value ≤0.05 was considered as statistically significant.

## Results

### Rotational behavior

**Figure 2:**
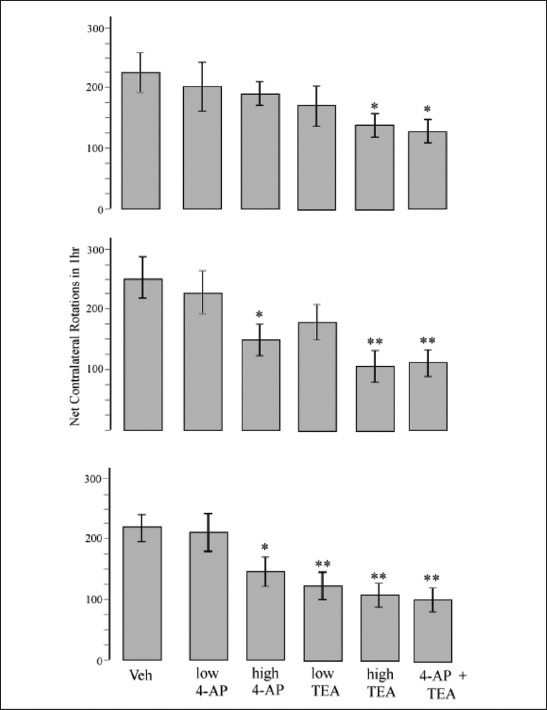
- Apomorphine- induced net contralateral rotations of different experimental groups at third (upper plot), fifth (middle plot) and eighth (lower plot) weeks post-surgery. Values are means±SE of each group. *p<0.05 and **p<0.01 compared to veh group, ^#^*p*<0.05 and ^##^*p*<0.01 compared to high 4-AP group, Kruskall–Wallis nonparametric test followed by Mann–Whitney U test. 4-AP - 4-aminopyridine, TEA - Tetraethylammonium, 6-OHDA - 6-hydroxydopamine, veh - 6-OHDA + saline

In all experimental groups, the rats showed various degrees of apomorphine-induced rotations, indicating that none of the pretreatments could prevent 6-OHDA-induced parkinsonism. However, as **Figure 2** shows, some of the pretreatments effectively ameliorated the development of parkinsonism. The 4-AP + TEA and high TEA groups showed the most powerful effects, whereby the number of net contralateral rotations was 40% less in the first test, 60% less in the second test, and 55% less in the third test than those observed in the veh group. Pretreatment with a high dose of 4-AP reduced the number of rotations by 36% in the second test and 32% in the third test. A low dose of TEA was also effective, but only meaningfully so in the third test.

### Swinging behavior

**Figure 3:**
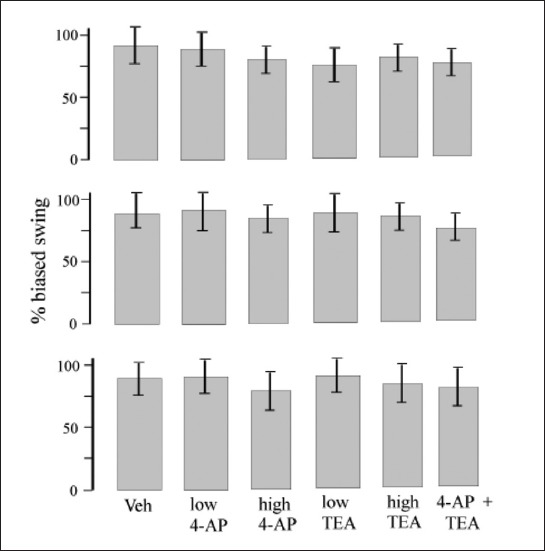
- Plots display results of the EBST in the third (upper plot), fifth (middle plot) and eighth (lower plot) weeks post-surgery. Values are means±SE of each group. 50% means number of left swings was equal to number of right swings. Less than 50% means that most of swings were toward left (contralateral to lesion side) and more than 50% means that most of swings were toward right side (ipsilateral to lesion side). *p<0.05 compared to veh group and ^#^*p*<0.05 compared to TEA groups, Kruskall–Wallis nonparametric test followed by Mann–Whitney U test. 4-AP - 4-aminopyridine, TEA - Tetraethylammonium, 6-OHDA - 6-hydroxydopamine, veh - 6-OHDA + saline

**Figure 3** shows the EBST test results. Pretreatment with TEA, 4AP + TEA and TEA did not change the degree and intensity of swings deviation, and no meaningful effects were observed in any of the groups.

### Rotarod test

**Figure 4:**
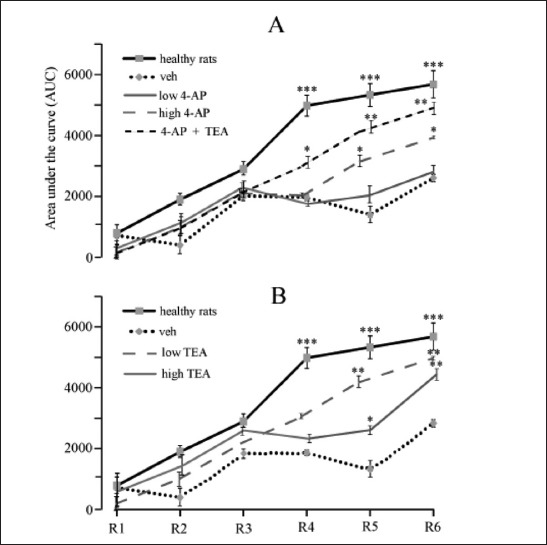
- Motor performance of different groups of rats in rotarod test examined at 3 consecutive days, 2 sessions in each. Healthy rats rapidly learned how to walk on the rotating rod and reached to maximum performance at fourth session. On the other hand, parkinsonian rats (veh) did not reach to maximum performance and showed much less learning. Learning pattern of motor performance in low TEA and 4-AP + TEA groups were very close to healthy rats while it was close to veh group in 4-AP groups and high TEA group. Values are means±SE of each group. *p<0.05, **p<0.01, ***p<0.001 compared to veh group, Kruskall–Wallis nonparametric test followed by Mann–Whitney U test. veh - 6-OHDA + saline AUC - area under the curve, for more description see the experimental procedures. R1-R6 - sessions of the test, R1 - first session, R6 - last session.

**Figure 4** shows the rotarod performance of all experimental groups. The motor performance of the healthy rats was better in every session, and peaked in sessions 4 to 6. The 6-OHDA-treated rats also showed different degrees of motor learning patterns, but significant differences were observed between these animals and the healthy rats. None of the 6-OHDA-treated group reached a normally expected performance, even in the final session. In addition, their learning pattern was different, and their performance did not improve in successive sessions. For example, walking time on the rotarod device was shorter in session 5 (R5) than in sessions 3 (R3) and 4 (R4) in the veh group. We examined the motor performance and learning pattern of rats in 2 sessions on each of the 3 successive days. The healthy rats rapidly learned how to walk and reached maximum performance at session 4. In contrast, the Parkinsonian rats in the veh group did not reach maximum performance, and showed far less learning. The motor performance patterns in the low TEA and 4-AP + TEA groups were very similar to those of healthy rats, while those observed in the 4-AP groups and high TEA group was similar to those of the veh group.

### MDA analysis

**Figure 5:**
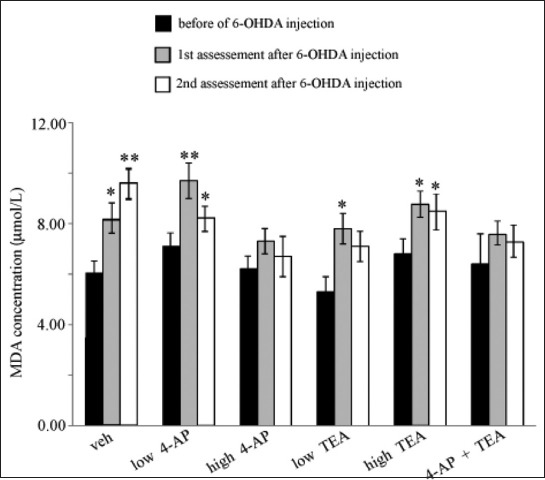
- Malondialdehyde (MDA) concentrations in sera of different experimental groups. *p<0.05, **p<0.01 compared to value before 6-OHDA injection, Kruskall–Wallis nonparametric test followed by Mann–Whitney U test. 4-AP - 4-aminopyridine, TEA - Tetraethylammonium, 6-OHDA - 6-hydroxydopamine, veh - 6-OHDA + saline

**Figure 5** shows the MDA concentrations in the sera of the different experimental groups. In the veh group, the MDA concentration was 6 ± 0.5 µmol/L before 6-OHDA injection, but this had increased by 26% at 3 weeks and 36% at 60 days after 6-OHDA injection. MDA concentrations increased in all groups after 6-OHDA injection, but decreased at the second assessment following injection. The reduction of MDA concentration was significant in the TEA+4-AP and high 4-AP groups.

## Discussion

The 6-OHDA-induced parkinsonism was created in 2 models: acute and chronic. The acute model was created by injection of 6-OHDA in the MFB of rat brains, which resulted in the death of dopaminergic neurons in the SN within 2 or 3 days. The chronic model was created by injection of 6-OHDA in the striatum of rat brains, which resulted in the death of dopaminergic neurons of the SN within 5 to 10 days. 6-hydroxydopamine was injected into the MFB instead of the striatum in order to mimic end-stage PD.^1^ Several reports have shown that there is a positive relevance between the death of SN dopaminergic neurons and the severity of behavioral symptoms in 6-OHDA-induced Parkinsonism.^13^,^15^,^16^ In addition, some studies have shown that there is a positive relationship between EBST and the apomorphine-induced rotational test in this model.^16^,^17^ Furthermore, it has also been shown that TEA exerts some beneficial effects in the treatment of several other diseases, such as multiple sclerosis,^18^,^19^ Alzheimer’s disease,^20^ and myasthenia gravis.^19^ The objective of the present study was to determine whether or not these K^+^-blockers are effective in the pretreatment of 6-OHDA-induced parkinsonism. The apomorphine-induced rotational test may lead to incorrect results as a result of desensitization to apomorphine; therefore, we evaluated the symptoms of parkinsonism using the EBST test.^17^ It has also been shown that the Rotarod test is one of the best with regard to evaluation of learning patterns and motor performance.^16^ Our results show that pretreatment with 4-AP and TEA significantly improved the behavioral symptoms of 6-OHDA-induced parkinsonism. TEA was effective at doses of both 2mg/kg and 5 mg/kg, but the lower dose was more powerful. A higher dose of 4-AP (1 mg/kg) was more effective than a dose of 0.5 mg/kg. In addition, administration of 4-AP and TEA combinations, as well as a low dose of TEA, significantly reduced rotation numbers and increased motor learning in the rotarod test. In order to evaluate the antiparkinsonian effect of 4-AP and TEA, we measured MDA concentration in rat sera, and we found that 6-OHDA increased MDA concentration. As a common biomarker of cellular oxidative stress, MDA is a product of fatty acids peroxidation.

We found that 4AP decreased MDA concentration, and least some of the antiparkinsonian effect of 4AP results from inhibition of oxidative stress. However, in rats that received TEA, MDA did not decrease significantly, indicating that the antiparkinsonian effect of TEA was not mediated by inhibition of oxidative stress. Several mechanisms can describe the neurotoxic effects of 4-AP and TEA on parkinsonism.

4-aminopyridine activates quiescent neurons via inactivation of A-type K^+^ channels, and triggers firing in numerous types of neurons. 4-aminopyridine also triggers high voltage-activated Ca^+^ channels, resulting in the strengthening of synaptic and neuromuscular transmission.^19^,^21^ Another mechanism is via 4-AP-induced activation of N-methyl-D-aspartate (NMDA) and non-NMDA glutamate receptors.^22^ TEA also activates silent neurons, augments neuronal firing frequency, and induces burst firing, as well as inhibiting large-conductance Ca2^+^-activated K^+^ channels.^23^

We found that glial cell line-derived neurotrophic factor (GDNF) may play an important role in the life and function of adult dopaminergic neurons, since the evidence shows that it is a powerful neurotrophic factor for growth, and dopaminergic neurons inhibit the resulting A-type K^+^ channel activity. 4-aminopyridine probably produced neuroprotective effects in this manner.

In conclusion, we provide evidence showing that pretreatment with the K^+^ channel-blockers 4-AP and TEA exerted an antiparkinsonian effect in a 6-OHDA-induced animal model. This effect was probably mediated by inhibition of oxidative stress, inhibition of the oxidant-induced K^+^ channel-dependent cell death pathway, and also via an activity-dependent mechanism that is involved in the control of dopaminergic neuron survival. Since we previously showed that 4-AP and TEA are also effective in the treatment of behavioral symptoms of 6-OHDA-induced parkinsonism, these blockers may be of use in the early stages of PD, to both reduce its symptoms and reduce SN dopaminergic cell death.
